# Graphene Oxide Framework Structures and Coatings: Impact on Cell Adhesion and Pre-Vascularization Processes for Bone Grafts

**DOI:** 10.3390/ijms23063379

**Published:** 2022-03-21

**Authors:** Fanlu Wang, Lena Marie Saure, Fabian Schütt, Felix Lorich, Florian Rasch, Ali Shaygan Nia, Xinliang Feng, Andreas Seekamp, Tim Klüter, Hendrik Naujokat, Rainer Adelung, Sabine Fuchs

**Affiliations:** 1 Experimental Trauma Surgery, Department of Orthopedics and Trauma Surgery, University Hospital of Schleswig-Holstein, Campus Kiel, Arnold-Heller-Straße 3, 24105 Kiel, Germany; fanluwang@googlemail.com (F.W.); felix@lorich.eu (F.L.); andreas.seekamp@uksh.de (A.S.); tim.klueter@uksh.de (T.K.); 2 Functional Nanomaterials, Institute for Materials Science, Kiel University, Kaiserstr. 2, 24143 Kiel, Germany; lms@tf.uni-kiel.de (L.M.S.); fas@tf.uni-kiel.de (F.S.); flce@tf.uni-kiel.de (F.R.); ra@tf.uni-kiel.de (R.A.); 3 Department of Chemistry and Food Chemistry, Center for Advancing Electronics Dresden (cfaed), Technische Universität Dresden, 01062 Dresden, Germany; ali.shaygan_nia@tu-dresden.de (A.S.N.); xinliang.feng@tu-dresden.de (X.F.); 4 Department of Oral and Maxillofacial Surgery, University Hospital of Schleswig-Holstein, Campus Kiel, Arnold-Heller-Straße 3, 24105 Kiel, Germany; hendrik.naujokat@uksh.de

**Keywords:** bone grafts, graphene oxide, vascularization, mesenchymal stem cells, VEGF

## Abstract

Graphene oxide (GO) is a promising material for bone tissue engineering, but the validation of its molecular biological effects, especially in the context of clinically applied materials, is still limited. In this study, we compare the effects of graphene oxide framework structures (F-GO) and reduced graphene oxide-based framework structures (F-rGO) as scaffold material with a special focus on vascularization associated processes and mechanisms in the bone. Highly porous networks of zinc oxide tetrapods serving as sacrificial templates were used to create F-GO and F-rGO with porosities >99% consisting of hollow interconnected microtubes. Framework materials were seeded with human mesenchymal stem cells (MSC), and the cell response was evaluated by confocal laser scanning microscopy (CLSM), deoxyribonucleic acid (DNA) quantification, real-time polymerase chain reaction (RT-PCR), enzyme-linked immunosorbent assay (ELISA), and alkaline phosphatase activity (ALP) to define their impact on cellular adhesion, osteogenic differentiation, and secretion of vascular growth factors. F-GO based scaffolds improved adhesion and growth of MSC as indicated by CLSM and DNA quantification. Further, F-GO showed a better vascular endothelial growth factor (VEGF) binding capacity and improved cell growth as well as the formation of microvascular capillary-like structures in co-cultures with outgrowth endothelial cells (OEC). These results clearly favored non-reduced graphene oxide in the form of F-GO for bone regeneration applications. To study GO in the context of a clinically used implant material, we coated a commercially available xenograft (Bio-Oss^®^ block) with GO and compared the growth of MSC in monoculture and in coculture with OEC to the native scaffold. We observed a significantly improved growth of MSC and formation of prevascular structures on coated Bio-Oss^®^, again associated with a higher VEGF binding capacity. We conclude that graphene oxide coating of this clinically used, but highly debiologized bone graft improves MSC cell adhesion and vascularization.

## 1. Introduction

Although bone tissue possesses an extraordinary self-healing ability, bone implants play an increasing role in the clinical routine. Critical-sized defects, defined as ones that would not heal spontaneously, demand for autogenous bone grafting or the use of a bone substitute material. Beyond ensuring mechanical stabilization, the main function of bone substitutes is to assist the cell-mediated bone healing processes after trauma- or disease- related bone loss. They act as substrates for new bone tissue formation and as osteoconductive scaffolds guiding these processes. In the clinical practice conventional bone graft materials classified as auto-, allo-, or xenografts play a major role among other groups of implant materials including metals, ceramics, polymers, and their composites [1].

Even though the native biological composition of autologous bone grafts including cells, growth factors, and components of the extracellular matrix supports the bone healing process, the use of autologous bone grafts is often restricted by a limited availability or quality of the bone tissue from the individual patient [2]. In comparison, the generation of allo- and xenograft-based implants includes severe chemical and physical processing steps to minimize risks of infection and immunological reactions [3,4,5]. These processing steps lead to the loss of bone-intrinsic biological factors essential for bone healing processes [6] and may further result in changes in terms of their biomechanical properties or their impact on stem cell functionality [7]. In general, the higher the processing-based security of the implants, the lower is their biological capacity. Therefore, the re-biologization of implants to support the process of osteoinduction is demanded in scaffold-based bone regeneration.

Biologization can be achieved by incorporating bioactive compounds, bone-related structural components, or regenerative cells to enhance bone healing in situ [8,9,10]. Further, the development of innovative materials and progress in versatile 3-D manufacturing and coating technologies to tailor bone scaffold design are essential when the dimensions of bone defects exceed a critical limit in size or complexity. The treatment of larger bone defects is very critical and has to consider functional blood vessel supply [11]. Bone formation and neovascularization are highly interconnected on the biological level and ensure the viability and functionality of the bone [12,13,14,15]. Further, both bone formation and vascularization request certain key features from an ideal implant design such as adequate pore size and a high interconnectivity [16,17].

In recent studies, the unique physiochemical properties of the graphene derivatives graphene oxide (GO) and reduced GO (rGO) have been reported for application in multiple areas, especially in tissue engineering and regenerative medicine [18,19,20].

GO coatings have further shown to enhance the functionality of bone implant surfaces such as improved osseointegration of laser-grooved titanium alloys [21], enabling a more biologized surface of this generally inert material. Further, GO has been integrated in poly(lactic-co-glycolic acid) (PLGA) electrospun fiber nets to enhance MSC functionality [20] by generating hydrophilic surfaces interacting with biological molecules and thus, facilitating cell material interactions. Recently, layer-by-layer technologies have been reported to create ultrathin films with graphene oxide and lysozyme for antibacterial coatings, limiting risks of infections in bone implants without interfering with their osteoinductive properties [22]. To generate antimicrobial surfaces, graphene oxide coatings have been further applied to build bilayer systems in combination with zinc oxide nanomaterials for the drug delivery of gentamicin sulfate [23], resulting in a more controlled release of the antibiotic compound over a wider period of time.

The objective of this study is to evaluate the impact of graphene oxide, with a special focus on cellular and molecular mechanisms guiding bone vascularization processes. In this context, graphene oxide F-GO and F-rGO were generated using highly porous (~94%) ZnO networks as a sacrificial ceramic template material [24]. First, we compared the impact of F-GO and F-rGO on human MSC cell adhesion and proliferation, as well as the expression of factors involved in osteogenic differentiation and vascularization. In functional studies, we assessed the influence of F-GO and F-rGO on the formation of microvascular structures in co-cultures of MSC with blood-derived outgrowth endothelial cells (OEC) [25,26]. Further, we tested the affinity of VEGF to the generated farmeworks.

To further evaluate graphene oxide coating in the context of a widely clinically used implant material we coated the bovine origin xenograft material Bio-Oss^®^ (Geistlich Biomaterials, Wolhousen, Switzerland) with GO and compared the biological activity of GO-coated samples with their native counterparts, as described above.

## 2. Results

### 2.1. Impact of F-GO and F-rGO on Mesenchymal Stem Cell Adhesion and Proliferation

**Figure 1:**
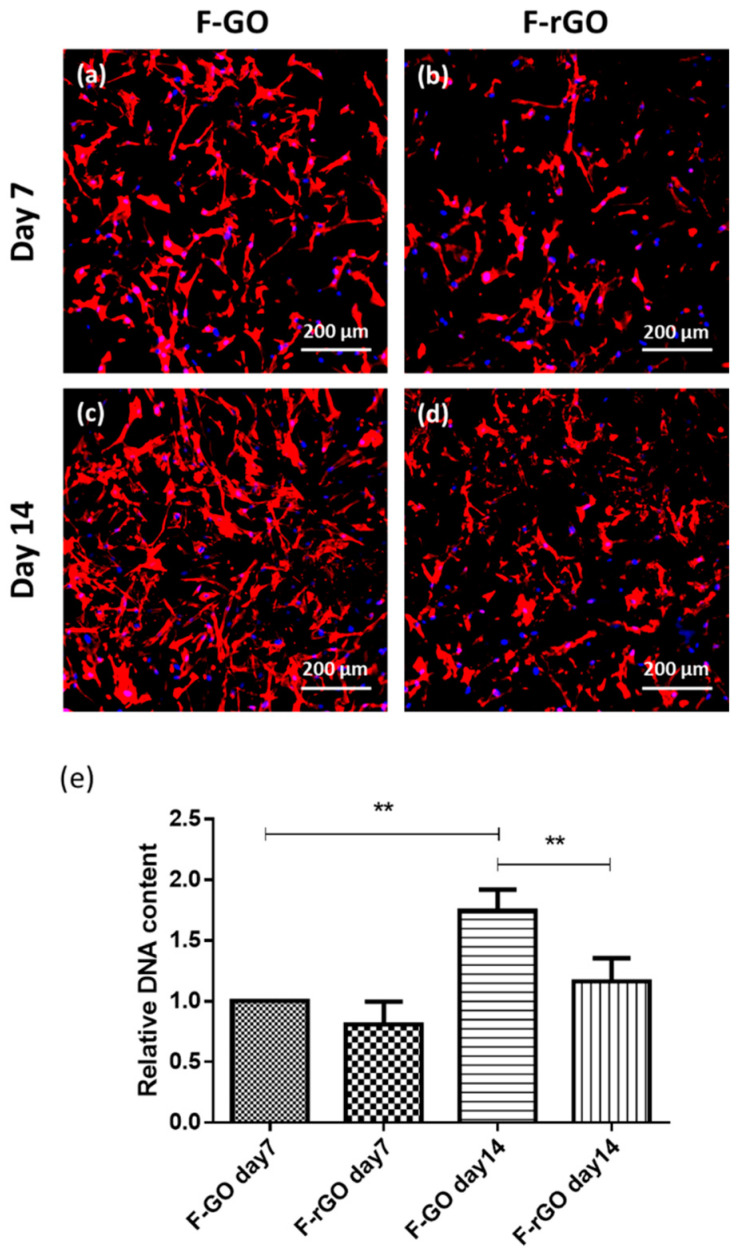
MSC growth on F-GO and F-rGO Frameworks. (**a**–**d**) MSC mono-cultures on F-GO or F-rGO on day 7 (**a**,**b**) and day 14 (**c**,**d**) visualized by confocal laser scanning microscopy after Phalloidin-TRITC staining. The scale bars represent 200 μm. (**e**) The relative DNA content of MSCs on framework structures on day 7 and day 14. One-way ANOVA, ** *p* < 0.01, *n* = 3 Donors.

GO reveals different properties depending on its state of oxidation. Thus, we first compared the impact of F-GO versus F-rGO. For this purpose, MSC were cultured on the surface of the framework networks, followed by analysis of cell adhesion and proliferation by CLSM and DNA quantification within a time course of 14 days. As indicated in Figure 1a–d, MSC adhered to both types of frameworks but showed higher cell densities on F-GO throughout the tested time frame (Figure 1a–d). This morphological observation by CLSM was further supported by quantitative DNA analysis, indicating a significantly higher proliferation rate and a significant increase in the DNA amount from day 7 to day 14 for F-GO (Figure 1e) for different MSC donors. In comparison, the DNA amount from MSC grown on F-rGO was significantly lower on day 14. Further, MSC differentiation markers were studied but showed only an increase in alkaline phosphatase (ALP) gene expression rates for F-rGO (Supplementary Figure S1).

### 2.2. Influence of F-GO and F-rGO on VEGF Levels and on VEGF Binding Rates

**Figure 2:**
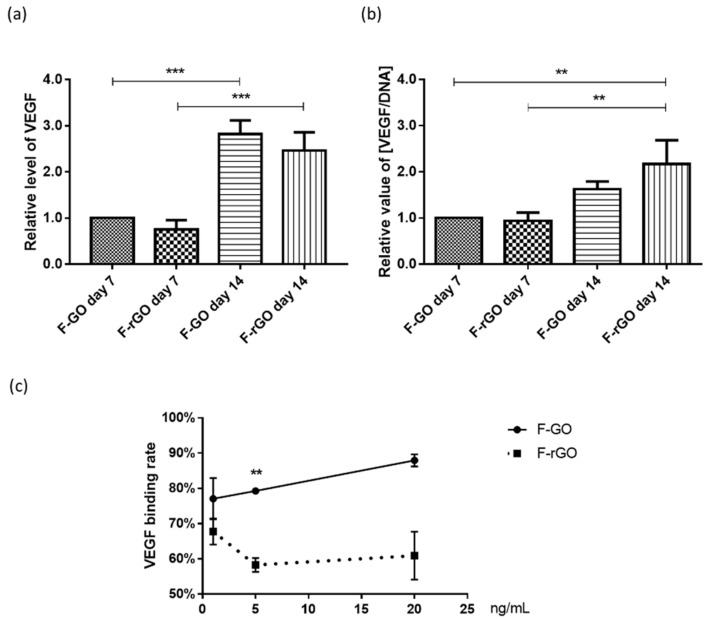
VEGF levels and binding capacities for F-GO and F-rGO. (**a**) Relative level of VEGF in the supernatant of MSC mono-cultures on F-GO or F-rGO on day 7 and 14, measured with enzyme-linked immunosorbent assay (ELISA). (**b**) VEGF level normalized to DNA content in relation to F-GO on day 7 for both time points. One-way ANOVA. ** *p* < 0.01, *** *p* < 0.001, *n* = 3 Donors of MSC. (**c**) VEGF binding rate for F-GO and F-rGO framework structures (in % of added concentration). *t*-test, ** *p* < 0.01, *n* = 3.

GO has been shown to interact with biological growth factors. These factors may naturally originate from cells in the tissue or might be added in the context of drug delivery approaches using implant materials. In this study, we focused specifically on vascularization processes. Thus, the levels of free VEGF in the supernatant of MSC grown on the frameworks were measured by ELISA. VEGF levels were depicted with and without normalization to the DNA content to evaluate influences by different cell numbers, as indicated by results in Figure 1e. Without normalization by the DNA content, VEGF levels showed the highest levels after 14 days of culture for GO samples (Figure 2a). However, with normalization, the VEGF levels were the highest for rGO on day 14 (Figure 2b). Nevertheless, no significant differences for VEGF levels in the supernatant between GO and rGO for MSC monocultures were observed on day 14, independent of the method of analysis.

In cell-free experiments, we analyzed the binding capacity of F-rGO and F-GO by adding defined VEGF concentrations of 1, 5, and 20 ng/mL in PBS (Figure 2c). We observed clear differences and a significant higher capacity to capture VEGF for F-GO at a concentration of 5 ng/mL.

### 2.3. Influence of F-GO and F-rGO on the Formation of Prevascular Structures Using Co-Culture Models

**Figure 3:**
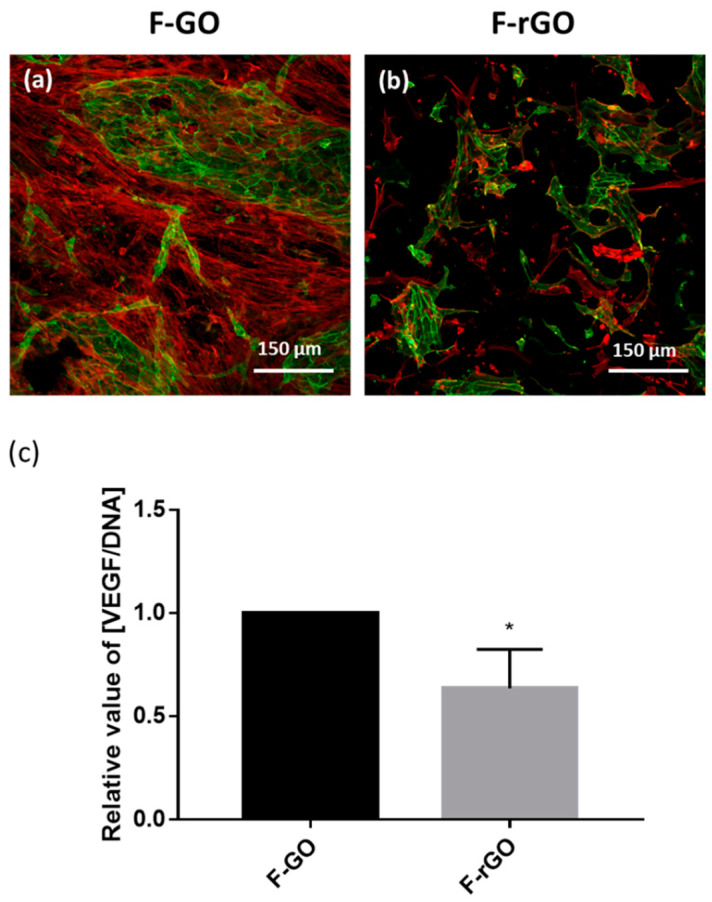
Impact of F-GO and F-rGO on vascularization processes. (**a**,**b**) MSC/OEC co-cultures on F-GO and F-rGO on day 7, visualized by CLSM. Cells were stained with VE-Cadherin (green), Phalloidin-TRITC (red) and Höchst (blue). The scale bars represent 150 μm. (**c**) The relative level of VEGF in the supernatant of MSC/OEC co-cultures on F-GO or F-rGO on day 7, measured with enzyme-linked immunosorbent assay. VEGF levels were normalized to DNA content and depicted in relation to F-GO (as control). *t*-test, * *p* < 0.05, *n* = 3 Donor Sets.

To assess if F-GO or F-rGO have a functional influence on the formation of prevascular structures, we co-cultured MSC and OEC on the surface of these framework materials. As indicated in Figure 3a,b, endothelial cells (depicted in green) grew better on F-GO samples compared to F-rGO counterparts. On F-GO, larger patches of endothelial cells and higher numbers of microvessel-like structure were observed. In contrast, F-rGO samples were characterized by a lower number of endothelial cells as well as lower numbers of MSC similar to the observations for MSC monocultures indicated before. Similar to these findings, the VEGF level in the supernatant normalized to DNA amount on F-rGO scaffolds was significantly lower (Figure 3c).

### 2.4. Graphene Oxide Coatings for Clinically Relevant Implant Materials

**Figure 4:**
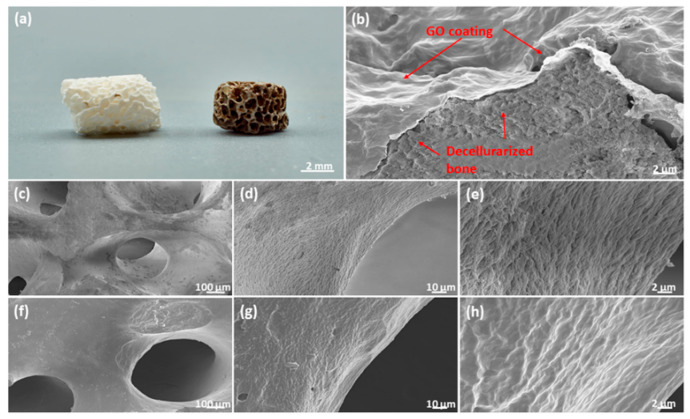
Graphene Oxide Coated Bio-Oss^®^. (**a**) Photograph of uncoated (left) and GO coated (right) Bio-Oss^®^ scaffold. (**b**) SEM micrograph of broken GO coated Bio-Oss^®^ scaffold with highlighted GO layer. (**c**–**e**) SEM micrographs of uncoated Bio-Oss^®^ scaffold in different magnifications, (**f**–**h**) SEM micrographs of GO coated Bio-Oss^®^ scaffold in different magnifications.

The data above suggested that non-reduced GO is preferable in terms of MSC adhesion and proliferation and has a beneficial influence on vascular processes. In the next step, we evaluated how GO coatings may affect the cellular response. For this purpose, a widely clinically applied bone graft material (Bio-Oss^®^) consisting of decellularized bone material was coated. The quality of the coating was documented by Scanning Electron Microscopy (SEM) (Figure 4a–h) and revealed a homogenous distribution of GO on the surface as well as along the porous structures of the implant. Further, GO coating changed the surface topography of the scaffold material by smoothing the implant surface (Figure 4b).

### 2.5. Impact of GO Coated Bio-Oss^®^ on MSC Function

**Figure 5:**
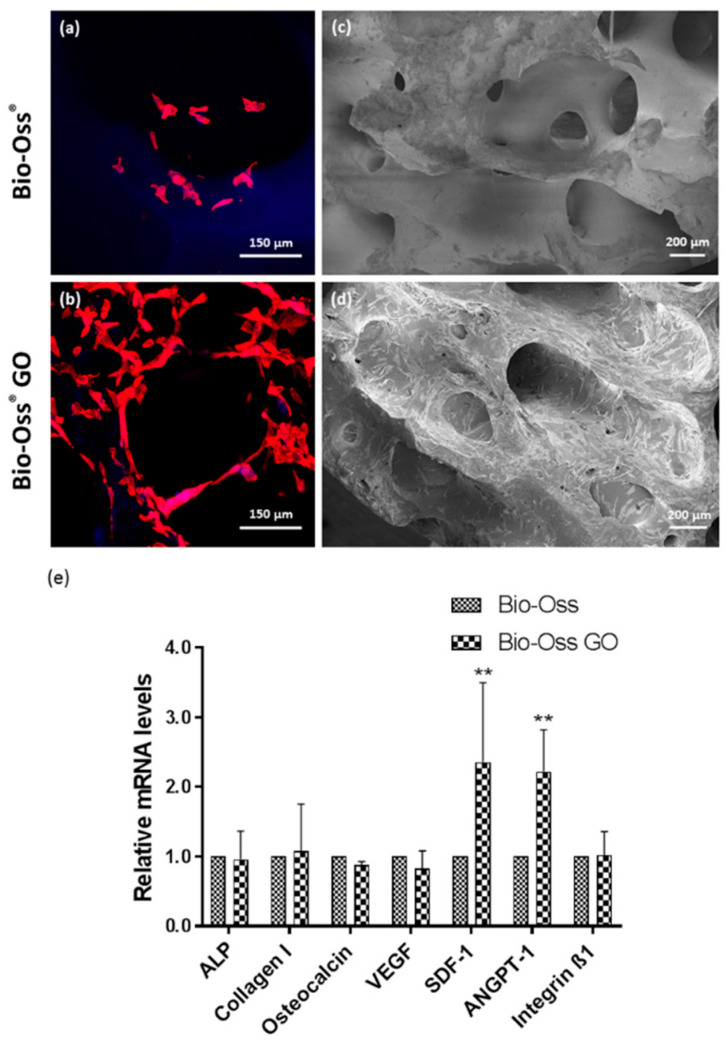
Growth of MSC on GO coated Bio-Oss^®^. (**a**,**b**) MSC mono-cultures on Bio-Oss^®^ (**a**) and GO coated Bio-Oss^®^ scaffolds (**b**) on day 7 visualized by confocal laser scanning microscopy. Cells were stained with Phalloidin-TRITC (red) and Höchst (blue). (**c**,**d**) SEM micrographs of MSC mono-culture on Bio-Oss^®^ (**c**) and GO coated Bio-Oss^®^ (**d**) scaffolds on day 7. (**e**) Relative gene expression of the osteogenic markers and cell-matrix adhesion molecule integrin evaluated by semi-quantitative RT-PCR for MSC mono-cultures on Bio-Oss^®^ and GO coated Bio-Oss^®^ scaffolds on day 7. Two-way ANOVA, ** *p* < 0.01, *n* = 3 Donors.

GO-coated Bio-Oss^®^ implants were compared with the native implants in terms of MSC performance (Figure 5). Morphological assessment revealed improved cell growth of MSC on the GO coated Bio-Oss^®^, as indicated by confocal microscopy (Figure 5b) and SEM (Figure 5d). Overall, MSC formed nearly confluent cell layers on the GO-coated Bio-Oss^®^ with cells also invading into the pores. In contrast, only single MSC attached to the non-coated Bio-Oss^®^ implants (Figure 5a,c).

To quantify the impact of the coating on MSC performance, we performed quantitative RT-PCR. The results indicated a significant impact of GO coating on the expression on stromal derived factor 1 (SDF-1) and angiopoitenin-1, factors involved in the recruitment of cells and vascularization during bone healing. In contrast, no significant influence on markers involved in osteogenic differentiation, such as collagen type-1 or osteocalcin, was observed (Figure 5e). DNA quantification to determine the difference in cell numbers in coated versus non coated samples was not feasible due to the high background in the DNA content originating from the decellularized Bio-Oss^®^ material (data not shown).

### 2.6. Impact of GO Coated Bio-Oss^®^ on Vascularization Processes

**Figure 6:**
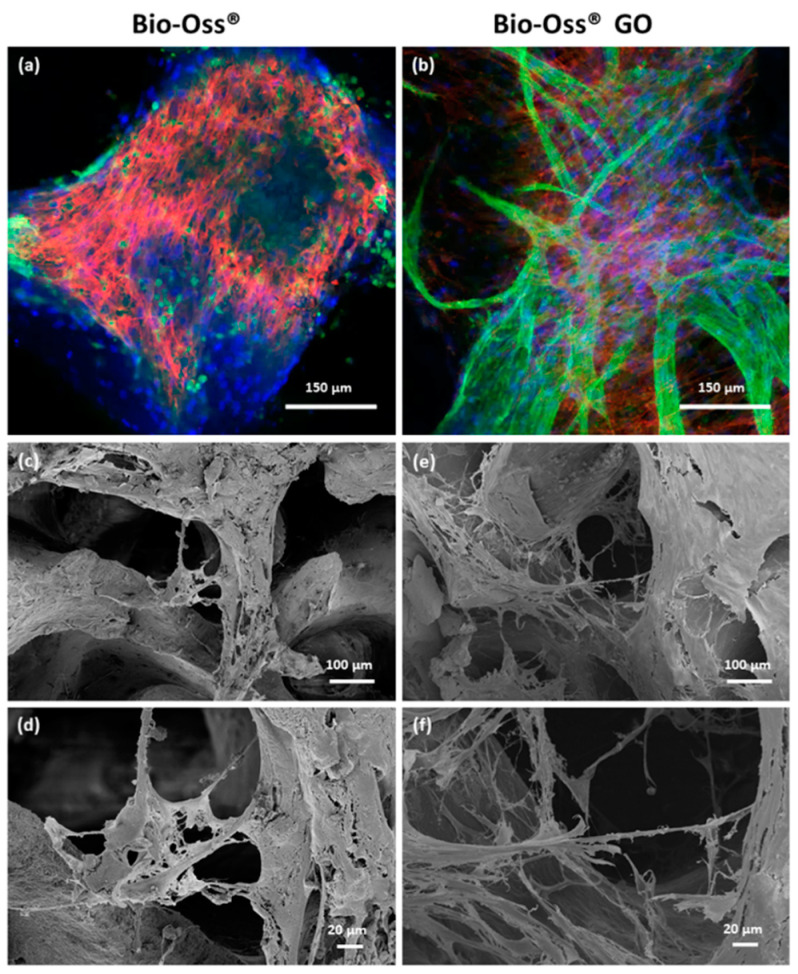
Morphological appearance of vascular structures on GO coated Bio-Oss^®^ (**a**,**b**) MSC/OEC co-cultures on Bio-Oss^®^ (**a**) and GO coated Bio-Oss^®^ scaffolds (**b**) on day 7 visualized by confocal laser scanning microscopy. Cells were stained with VE-Cadherin (green), Phalloidin-TRITC (red) and Höchst (blue). (**c**–**f**) SEM micrographs of MSC/OEC co-cultures on Bio-Oss^®^ (**c**,**d**) and GO-coated Bio-Oss^®^ (**e**,**f**) scaffolds on day 7.

In the next steps, we focused on analyzing effects on vascularization for GO coated Bio-Oss^®^ in comparison to the native Bio-Oss^®^ material. Co-cultures of MSC and OEC on GO-coated Bio-Oss^®^ revealed larger areas of coherent endothelial cell layers, as well as distinct and complex prevascular structures as indicated by CLSM (Figure 6b) and SEM (Figure 6e,f) analysis. The porous structure of the bone implant was maintained after the coating process facilitating the ingrowth of cells along the pores of the scaffold. These observations were consistent for different donor sets. In comparison, non-coated Bio-Oss^®^ samples revealed fewer cells in total, independent of the cell type, as well as only occasionally occurring vascular structures with a much lower complexity (Figure 6a,c,d).

### 2.7. Quantitative Assessment of Cellular Markers and Vascularization Associated Factors on GO Coated Bio-Oss^®^

In the SEM analysis, differentiation between MSC and endothelial cell types is only feasible to a certain extent based on morphological cell features. Nevertheless, SEM micrographs can be compared to data gained by immunofluorescence based on cell-type-specific staining procedures. To underline the better performance of the GO-coated Bio-Oss^®^ with quantitative data, we performed semi-quantitative real-time PCR.

**Figure 7:**
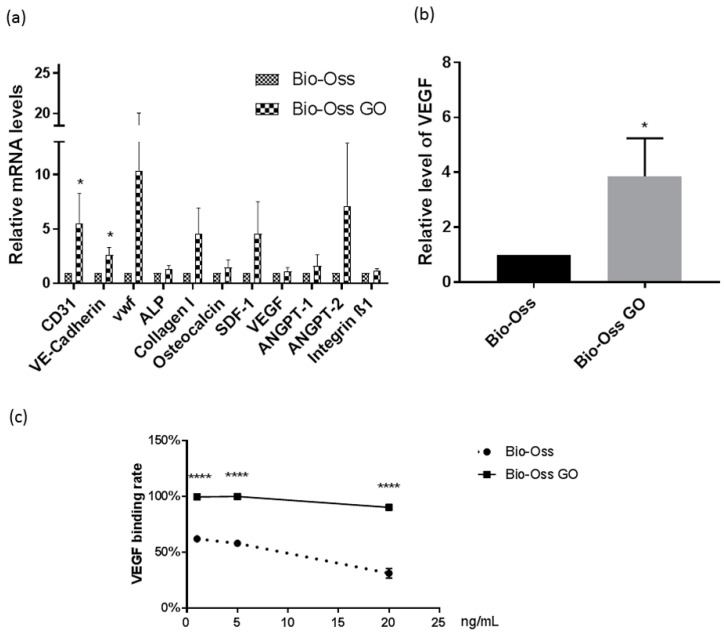
Quantitative evaluation of angiogenesis related markers on GO coated Bio-Oss^®^. (**a**) Relative gene expression of the endothelial markers, osteogenic markers, and cell–matrix adhesion molecule integrin evaluated by semi-quantitative RT-PCR for MSC/OEC co-cultures on Bio-Oss^®^ and GO coated Bio-Oss^®^ scaffolds on day 7. Two-way ANOVA, * *p* < 0.05, *n* = 3 donor sets. (**b**) VEGF in supernatant of MSC/OEC co-cultures on Bio-Oss^®^ or GO coated Bio-Oss^®^ on day 7 in relation to Bio-Oss^®^ (as control). *t*-test, *n* = 3 donor sets. (**c**) VEGF binding to the Bio-Oss^®^ scaffolds w/wo GO coating (in %VEGF to control solution). *t*-test, **** *p* < 0.0001, *n* = 3.

RT-PCR verified the morphological observations in terms of vascularization on the quantitative level showing tentatively or significantly higher gene expression for endothelial markers (CD31, VE-Cadherin, vWF) and growth factors produced by endothelial cells (ANGT-2) (Figure 7a). Although the impact on osteogenic differentiation markers was less evident, collagen type 1, as a marker for early osteogenic differentiation, was tentatively increased but without statistical significance.

Coherent with a better attachment of MSC, the main producer of VEGF, we observed significantly elevated VEGF levels in the supernatant for co-cultures grown on GO coated Bio-Oss^®^ (Figure 7b).

Furthermore, we evaluated the impact of GO coating on the VEGF binding capacity of Bio-Oss^®^ in cell free experiments, as reported before (Figure 7c). We observed a significantly higher VEGF binding capacity for all tested VEGF concentrations (1, 5, and 20 ng/mL) for GO-coated Bio-Oss^®^. This higher capacity to bind VEGF by the GO coating improves the angiogenic potential of Bio-Oss^®^.

## 3. Discussion

In this study, we compared the effect of GO and rGO on bone regeneration processes. Experiments ranged from analyzing the adhesion and proliferation of human MSC to the assessment of prevascular structures in co-cultures of MSC and OEC mimicking bone vascularization processes. The impact of GO and rGO was investigated on the mechanistic level, taking into account cell and molecular biological processes and associated mediator molecules in vascularization and bone formation.

We have shown that particular frameworks consisting of pure GO (F-GO) or GO as coating of clinically relevant Bio-Oss^®^ grafts support the growth of MSC and further enhance the formation of prevascular structures. Prevascular structures for both F-GO frameworks and GO-coated Bio-Oss^®^ were complex and characterized by tube-like structures as indicated by CLSM and SEM.

The solid impact of GO on endothelial cells, as observed on the morphological and functional level, was further supported by the quantitative evaluation of endothelial markers widely increasing on GO-coated Bio-Oss^®^ xenografts. We have further shown that GO as a structural element in scaffolds or as a coating material increases the capacity to bind VEGF, representing one of the potential mechanisms how GO supports vascularization-associated processes. The binding of VEGF by GO has also been reported by other groups, although in the context of anti-angiogenic applications for tumor treatment [27].

The results of our study are in accordance with the present knowledge regarding properties of GO, revealing numerous oxygen-containing groups, such as hydroxyl, epoxy, and carboxyl groups [28]. These functional groups enhance the hydrophilicity of the GO surface and enable functionalization of GO with bio-active molecules [29] and interaction with a series of proteins. In vivo, these proteins may originate from blood serum [30], whereas in biotechnological approaches, distinct proteins might be chosen to generate bio-artificial or bio-mimetic surfaces of implants [31]. GO surfaces offer a broad spectrum for surface modification [32]. Besides using individual proteins, modification may include total antibodies or DNA fragments, but also offer the possibility to use different principles in surface-modifications, either based on directed bio-conjugation or physicochemical adsorption [32].

The reduction of functional groups in GO results in an alteration of the physiochemical properties in the reduced form (rGO), such as surface properties and conductivity [33]. Although the reduction process from GO to rGO leads to a lower number of oxygenated groups, not all of them might be affected. The reduction process may result in some variations also affecting the biological consequences. Nevertheless, our results were consistent throughout the different methods used to evaluate the impact of GO and rGO on the biological level and included several batches of materials.

GO is often used in combination with a variety of other materials to generate scaffolds and bone implants [18,20,34], for instance, in form of composites [35,36] or to reinforce bone cements [37,38]. In the first part of our study, we focus on the comparison of frameworks consisting of GO or rGO. The fabrication of these GO and rGO framework structures was implemented by using highly porous networks of ZnO tetrapods as sacrificial and highly nano porous template structure. After the removal of ZnO, only GO or rGO remained. Accordingly, the influences of other material components on the cell response can be widely excluded in the frameworks, allowing us to compare the distinct impact of GO or rGO on a detailed biological level. Data were reproducible in complex co-cultures of MSC and OEC gained from various donors, further underlining the reliability of the effects in a complex biological context.

ZnO has been applied in bone tissue engineering as a building material for bone implants in combination with GO for drug delivery [23]. Recently, ZnO tetrapods have been used as a sacrificial material to create highly porous templates for infiltration with carbon nanotubes and bioactive nanoparticles based on bioactive glass and hydroxyapatite [39] This approach has been suggested as a new and versatile technology to create 3D implants for bone tissue regeneration. The resulting highly porous hybrid scaffolds allowed the adhesion of osteogenic cell lines on the surface of these scaffolds. In our study, the frameworks consisted of GO or rGO only but revealed a similar porous structure and a high compatibility for human MSC and blood-derived endothelial cells for the F-GO.

Despite the fact that the F-GO favored the attachment and proliferation of MSC as well as the formation of vascular structures on the implant surface, the F-GO resulting from the ZnO templating approach lack the mechanical stability essential for bone implants. Further, larger pores and a highly interconnected scaffold architecture are essential for bone substitute materials and particularly necessary to ensure bone regeneration and vascularization in critical-sized bone defects. Thus, mechanical stability of the demonstrated framework structures should be adapted in future experiments using a combination of GO with polymers or hydroxyapatite, along with additional technologies to increase the pore size.

Based on the findings showing the beneficial impact of GO on MSC adhesion, proliferation, and vascularization, we decided to proceed using GO to coat the clinically relevant xenograft material Bio-Oss^®^. Bio-Oss^®^ is derived from bovine bone and is physically and chemically highly processed. On one hand, the processing is a must to limit infection and immunological risks. Nevertheless, the removal or denaturation of proteins and signaling molecules might interfere with the osteoinductivity. Although Bio-Oss^®^ is widely used in the clinic, resulting in bone formation in vivo, the knowledge of its impact on individual cell response is quite limited and may even depend on the size when granular forms of Bio-Oss^®^ are applied, influencing inflammation and vascularization [40]. Nevertheless, Bio-Oss^®^ as an implant provides all architectural features of the human bone spongiosa [41].

On native Bio-Oss^®^ without GO coating, MSC adhesion in this present study was characterized by single-cell adhesion, similar to a previous study from our group [7]. We assume that the high grade of de-biologization such as the removal of proteins leaving behind only the mineral content in Bio-Oss^®^ is a critical factor interfering with MSC adhesion. Scaffolds only consisting of the bone mineral phase reveal drawbacks in their interaction with cells and vascularization, as shown before [42,43].

GO coating, on the other hand, improved the adhesion of MSC significantly, along with a smoother surface of the implant, as indicated by CLSM and SEM. However, this was investigated at day 7 of the culture, not in earlier phases, to cope with the focus on vascularization in this present study. Further, the coating procedure improved the endothelial cell performance in the cocultures, as indicated on the morphological level by CLSM and SEM and by revealing a quantitative increase in endothelial markers in the real-time PCR data. In initial in vitro studies, we observed no increase in interleukin-6 levels, an indicator of inflammatory activation of endothelial cells, after coating Bio-Oss^®^ with GO (cocultures day 7, data not shown). However, inflammatory reactions are complex, and in this study, reactions of immune cells were not included.

The impact of GO on angiogenesis in this study should be considered as a multifactorial process. Although a higher capacity to bind VEGF of GO coated Bio-Oss^®^ is one important mechanism supporting the vascularization process, simply a better adhesion and growth of MSC supports the vascularization and performance of OEC due to their paracrine interaction [44,45,46]. MSC produce high levels of VEGF, and their direct interaction with endothelial cells is a key element in bone formation and vascularization. This mechanism is also reflected in the ELISA data from the supernatants from GO coated Bio-Oss^®^ compared to the native implant revealing significantly increased VEGF levels.

Coating Bio-Oss^®^ using PLA (Poly-D,L- lactic acid) [47] or PLCL (Polycaprolacton) [48] has been shown before to improve the cell growth of osteogenic cell lines or MSC in bone implants, whereas GO coating has not yet been reported for Bio-Oss^®^ to our best knowledge.

## 4. Materials and Methods

### 4.1. Isolation and Culture of Human Mesenchymal Stem Cells (MSCs)

MSCs were isolated from bone fragments of a human femoral head, as previously described [44]. In brief, the bone marrow cells were collected in tissue buffer (Medium 199, GlutaMax^TM^ (Gibco, Darmstadt, Germany), 20% fetal bovine serum (FBS) (Sigma, Taufkirchen, Germany), 1% penicillin/streptomycin (Pen/Strep) (Biochrom, Berlin, Germany), 1% fungizone (Biozol, Eching, Germany), and 1% ciprobay (FRESENIUS KABI, Bad Homburg Germany)) by washing cancellous bone fragments, to which the cells loosely attach. After centrifuging at 400 g for 5 min, cells were resuspended in growth medium (Dulbecco’s Medium Essential Medium (DMEM)/Ham F-12 (Biochrom, Berlin, Germany) supplemented with 20% FBS and 1% Pen/Strep) and seeded to tissue culture flasks coated with collagen I (Corning, Bedford, MA, USA) at a density of 2 × 10^6^ cells/cm^2^. Osteogenic differentiation was induced in ODM (osteogenic differentiation medium, DMEM/Ham F-12; 0.1 μM dexamethasone (Sigma-Aldrich, St. Louis, MO, USA); 10 mM β-glycerol phosphate (Sigma-Aldrich); and 50 μM ascorbic acid-2-phosphate (Sigma-Aldrich) 10% FBS, and 1% Pen/Strep) upon passage 2 for at least 14 days before the experiments.

### 4.2. Isolation and Culture of Human Outgrowth Endothelial Cells (OECs)

OECs were isolated from human blood according to protocols previously described [25,26]. First, human mononuclear cells were isolated from buffy coat by gradient centrifugation with Biocoll (Biochrom, Berlin, Germany) and collected in Endothelial Cell Growth Medium (ECGM-2) (PromoCell, Heidelberg, Germany) with supplements from the kit, 5% FBS and 1% Pen/Strep. Cells were seeded to collagen I-coated 24-well plates at a density of 5 × 10^6^ cells/well and subcultured to new plates at a density of 5 × 10^5^ cells/well after 7 days. After 2–3 weeks’ culture, cobblestone-like OEC colonies appeared and were expanded for the co-cultures.

### 4.3. Fabrication of Graphene Oxide and Reduced Graphene Oxide Framework Structures

F-GO and F-rGO were fabricated using highly porous (~94%) sacrificial ceramic templates composed of interconnected ZnO microparticles with tetrapodal shape, as described in more detail elsewhere [24,49]. In brief, tetrapodal ZnO (t-ZnO) powder was synthesized by the flame transport synthesis (FTS) [50,51,52,53], where zinc powder and polyvinyl butyral was mixed in the weight ratio 1:2 and heated in muffle furnace to 900°C for 30 min with a heating rate of 60 °C/min. A defined amount of t-ZnO powder was pressed into templates with cylindrical shape (9.5 mm diameter, 2 mm height) using a metal mold. Annealing at 1150 °C for 5 h resulted in a freestanding macroscopic network of interconnected ZnO tetrapods with a density of 0.3 g cm^−3^ (corresponding to a porosity of 94%).

The open–porous structure allows for a wet-chemical infiltration with a water-based dispersion of GO, which was produced as reported elsewhere [54,55] and dispersed in water via tip sonication. The GO dispersion (0.2 wt% graphene oxide flakes in water) was dribbled onto the templates until the free volume was filled completely, followed by drying of the samples on a heating plate at 50 °C. During evaporation of the solvent, the GO sheets form a homogenous thin (<25 nm) layer on the ZnO surface [49]. The infiltration process was repeated four times, resulting in a homogenous coverage of GO flakes on the ZnO surface. Subsequent wet-chemical removal of ZnO with 1 M hydrochloric acid (HCl) resulted in freestanding F-GO composed of interconnected hollow microtubes. After wet-chemical etching, the samples were washed thoroughly in water (3×) and absolute ethanol (5×) and dried using a critical point dryer (EMS 3000).

**Figure 8:**
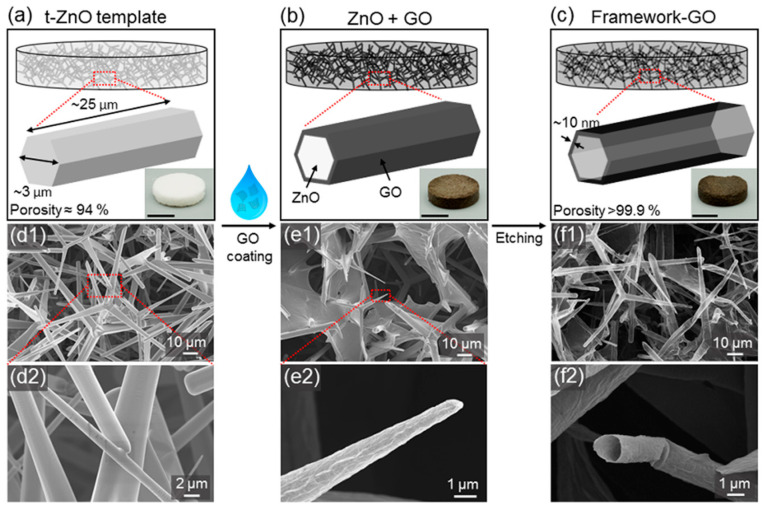
Flow chart of fabrication of graphene oxide framework structures (F-GO). Fabrication steps of graphene oxide framework structures (F-GO): (**a**) schematic of wet chemical assembly of GO into interconnected microtubular structure. (**a**) Sacrificial templates of tetrapodal ZnO are infiltrated with aqueous dispersion of GO flakes resulting in (**b**) a homogeneous coverage of individual ZnO microrods with GO. (**c**) Wet-chemical etching of ZnO leads to a freestanding framework structure of hollow microtubes consisting of GO with a porosity of more than 99%. The insets show photographs of a sample in each production step. Scale bars: 5 mm. (**d**–**f**) SEM micrographs of (**d**) network of tetrapodal ZnO, (**e**) ZnO tetrapods covered with GO, and (**f**) framework structure of hollow GO microtubes.

F-rGO were fabricated according to the aforementioned method with an additional reduction step prior to the wet-chemical etching of ZnO, during which the GO is reduced using L-ascorbic acid diluted in water (0.1 mg mL^−1^). The fabrication process and main structural features of the framework structures are depicted as schematic overview, as well as in scanning electron microscopy (SEM) micrographs in Figure 8a–f.

### 4.4. GO Coating of Hydroxyapatite Xenografts

In this study, commercially available cancellous xenografts of bovine origin (Bio-Oss^®^ blocks, Geistlich Pharma AG, Wolhusen, Switzerland) served as source for the hydroxyapatite scaffolds. Bio-Oss^®^ material was selected due its high grade of de-biologization during the manufacturing process including the removal of proteins followed by sintering at high temperatures and due to its high biocompatibility in preclinical and clinical studies [8,9,10]. The blocks present a favorable interconnected porosity, and the spongious trabecular structure of the donor-bone is preserved. In brief, the scaffolds were drilled out from the blocks (1 × 1 × 2 cm) into Ø 6 mm × 5 mm cylinders with a table drilling machine (Bosch, PBD40, Germany) and coated with a graphene oxide layer. In detail, cylindrical samples of Bio-Oss^®^ were immersed in aqueous GO dispersion (0.2 wt%), prepared as described before, and infiltrated at low vacuum using a desiccator to fill the pores of the scaffold with GO dispersion. Subsequent drying on a heating plate (50 °C) resulted in a homogeneous coating of GO on the surface of the scaffolds. The coating process was performed twice.

### 4.5. Cell Seeding on the Scaffolds

#### 4.5.1. GO/rGO Framework Structures

The F-GO and F-rGO were autoclaved and incubated in 1 mL ODM per sample for 24 h before seeding cells. Human mesenchymal stem cells (MSCs) were seeded onto the sample at a density of 100,000 cells per scaffold in 1 mL ODM in an agarose (1%, Invitrogen, Carlsbad, CA, USA) -coated 48-well plate. The medium was changed every other day.

For OEC/MSC co-cultures, outgrowth endothelial cells (OECs) were seeded to the scaffolds the next day at the same density in ECGM-2 and fed with fresh medium every second day.

#### 4.5.2. Bio-Oss^®^ and GO-Coated Bio-Oss^®^

The bone scaffolds (Bio-Oss^®^ and the GO coated Bio-Oss^®^) were prepared according to the method in 2.5 and by 20 min incubation in 70% Ethanol (Chemsolute, Renningen, Germany) followed by 3 × PBS wash and then incubated in ODM for 24 h. Human mesenchymal stem cells (MSCs) were seeded to the scaffolds at a density of 150,000 cells per scaffold in 750 µL ODM in an agarose (1%) -coated 48-well-plate. For co-cultures, OECs were seeded the next day at the same density after MSCs. The MSC mono-cultures were kept in ODM, and co-cultures were cultivated in ECGM-2. Medium change was performed every second day.

### 4.6. Immunofluorescence Staining and Visualization

Cells on the scaffolds were fixed on day 7 with 4% paraformaldehyde (PFA) in PBS (Affymetrix, Cleveland, OH, USA) for 15 min and permeabilized with 0.05% Triton^®^X-100 (in PBS, Sigma-Aldrich, Taufkirchen, Germany) for 10 min. For MSCs mono-cultures, cells were stained with Phalloidin-TRITC (1:100 in PBS, Sigma-Aldrich) for 30 min and then Höchst (2 µg/mL in PBS, Sigma-Aldrich) for 10 min. The co-cultures were incubated with VE-Cadherin (R&D, Minneapolis, MN, USA) antibodies (1:50 diluted in PBS with 1% bovine serum albumin (BSA)) for 2 h. After being washed three times with PBS, the cells were incubated with the secondary antibody (1:1000 in 1% BSA) and Phalloidin-TRITC (1:100) for 45 min, followed by 10 min incubation with Höchst (2 µg/mL in PBS). The immuno-stained samples were kept in PBS for visualization with confocal laser scanning microscope (CLSM, LSM 800, Zeiss, Germany) affiliated with ZEN image system.

### 4.7. Scanning Electron Microscopy (SEM)

Cells on the scaffolds were fixed with 3% glutaraldehyde (Sigma-Aldrich) in PBS for 2 h. After washing with PBS, the samples were incubated in ethanol (AppliChem, Darmstadt, Germany) gradients from 50 to 99% and stored in 99.99% ethanol and dried with a critical point dryer (EMS 3000) for scanning electron microscopy (SEM). The samples were glued onto sample holders with conductive carbon tape and sputtered with Au for 90 s. SEM characterization was performed using a Zeiss Supra 55VP.

### 4.8. Quantification of DNA Content

To collect DNA samples from F-GO/F-rGO, the samples were washed with PBS and transferred to 1 mL nuclease-free water for each sample. The cell membrane was ruptured using 3 freeze–thaw cycles, and DNA samples were prepared by further ultrasonic treatment.

The DNA content was examined with Quant-iT PicoGreen dsDNA assay kit (Molecular probes, Eugene, OR, USA). Samples and standards were prepared in triplicates, and DNA amount was determined by fluorescence using a microplate reader (TECAN, Maennedorf, Switzerland) at 485/535 nm of excitation/emission wavelength according to a standard curve.

### 4.9. Gene Expression Analysis

The constructs were washed with PBS, and cells were lysed in TRIzol^TM^ (ambion, Carlsbad, CA, USA). After 3 freeze–thaw cycles, the TRIzol solution from each group was collected into a new microtube and vigorously mixed with chloroform (200 μL per mL TRIzol solution, MERCK, Darmstadt, Germany). After centrifugation at 12,000 g for 15 min at 5 °C, the upper colorless aqueous phase was collected to perform the total RNA isolation (peqlab, Germany) according to the manufacturer’s protocol. The RNA concentration was measured with a NanoDrop (Thermo Fisher, Erlangen, Germany) and transcribed to cDNA with high-capacity RNA-to cDNA Kit (Applied Biosystems, Carlsbad, CA, USA) using 1 μg for each sample. Real-time PCR was implemented for the primers shown in Table 1, with RPL13a as internal control. A 20 μL total volume reaction mixture, which consisted of the SYBR^®^ Select Master Mix (applied biosystems, Austin, USA), cDNA QuantiTect^®^ Primer Assay (Qiagen), RNase free water (Qiagen), and cDNA, was prepared in duplicate for each sample. The mixtures were preheated to 50 °C for 20 min and 95 °C for 20 min followed by 40 cycles of step 1, 95° for 15 sec, and step 2, 60° for 60 sec. The relative gene expression was calculated with ΔΔcT method with control referenced as 1.

### 4.10. Quantification of Osteogenic Activity

To determine the osteogenic activity of MSCs on the constructs, the culture supernatants were collected on day 7 and 14 to perform alkaline phosphatase (ALP) activity assay with the Alkaline Phosphatase Assay Kit (Colorimetric) (Abcam, Germany) according to the manufacturer’s protocol. In brief, the samples and standards were prepared in triplicate and applied to 96-well plates for ALP reaction with pNPP solution. Before the ALP reaction was initiated, the background control was prepared by adding stop solution. After 60 min incubation, the reaction was inactivated by stop solution for the samples and standards. The OD value was measured at 405 nm with Apollo reader. The absorption of background control was subtracted for calculation of ALP activity, and the results were presented in relation to the control group.

After 14 days’ cultivation, the constructs were washed with PBS, and cells were fixed with 4% PFA in PBS followed by Alizarin Red staining (Millipore, Billerica, MA, USA) for 30 min. The excess dye was completely removed by distilled water, and the Alizarin Red dyes bound to the mineralized extracellular matrix were extracted into 10% (*w*/*v*) cetylpyridinium chloride (CPC) (Roth, Karlsruhe, Germany). The sample Alizarin Red solution and standards prepared in 10% CPC were added to a 96-well plate and read at 560 nm in a microplate reader (Apollo) for quantitative analysis.

### 4.11. Enzyme Linked Immunosorbent Assay (ELISA)

The VEGF level in the supernatant was determined with DuoSet ELISA Development kit (R&D, Minneapolis, MN, USA) according to manufacturer’s protocols. In brief, a 96-well plate (Greiner Bio-One, Germany) was coated with capture antibodies (100 µL/well, diluted in PBS) and incubated at room temperature overnight. The coated plate was washed with wash buffer (0.05% Tween^®^ 20 in PBS) to remove the excess capture antibodies and blocked with reagent dilute (1% BSA in PBS, filtrated) for 1 h. After washing, samples and standards were prepared in triplicate and applied to the plate for 2 h incubation at room temperature. The detection antibodies diluted in 1% BSA in PBS were applied to the plate after washing and incubated for 2 h at room temperature, followed by the colorimetric reaction using Streptavidin-HRP conjugates. The optical absorbance was detected by the microplate reader (Apollo) at 450 nm, with a reference wavelength of 560 nm. VEGF levels were presented in relative values compared to controls.

### 4.12. VEGF Binding Capacity of F-GO and F-rGO or GO Coated Bio-Oss^®^

hHuVEGF-165 solutions (R&D, Minneapolis, MN, USA) of 1, 5, and 20 ng/mL were prepared in order to obtain the VEGF binding curve to various materials at different concentrations. First, all scaffolds were hydrated in PBS for 24 h and then incubated in VEGF stock solution for 48 h at 37 °C. Both stock and solutions incubated with scaffolds were collected for determine VEGF level with DuoSet ELISA Development kit. Samples and standards were prepared in triplicates. The amount of bound VEGF was calculated and presented in percentage to the corresponding stock solution (as control).

### 4.13. Statistics

All experiments mentioned above were performed with cells of 3 different donors/donor combinations. The statistical significance was assessed as shown for each graph with *t*-test or ANOVA using Graphpad Prism 7, as indicated in the corresponding result sections; *p* < 0.05 (* *p* < 0.05, ** *p* < 0.01, *** *p* < 0.001, **** *p* < 0.0001) was considered as statistically significant.

## 5. Conclusions

In conclusion, by directly comparing the influence of GO- and rGO-based framework structures, data regarding bone cell adhesion and vascularization processes favored, in our study, GO over rGO for bone regeneration applications.

Due to the interaction of GO with proteins, in this study, represented by the binding of VEGF, coatings with GO could be a highly useful technology, especially for allo- and xenografts, which lost intrinsic biologic factors during the processing steps, and thus provide a platform to modify these clinically highly relevant materials.

## Figures and Tables

**Table 1:** List of primers.

Gene Name	Primer Assay	Catalogue Number
ALP	Hs_ALPL_1_SG QuantiTect Primer Assay	QT00012957
CD31	Hs_PECAM1_1_SG QuantiTect Primer Assay	QT00081172
Osteocalcin	Hs_BGLAP_1_SG QuantiTect Primer Assay	QT00232771
Angiopoietin-1	Hs_ANGPT1_1_SG QuantiTect Primer Assay	QT00046865
Angiopoietin-2	Hs_ANGPT2_1_SG QuantiTect Primer Assay	QT00100947
Collagen type I	Hs_COL1A1_1_SG QuantiTect Primer Assay	QT00037793
SDF-1	Hs_CXCL12_1_SG QuantiTect Primer Assay	QT00087591
VE-Cadherin	Hs_CDH5_1_SG QuantiTect Primer Assay	QT00013244
VEGF	Hs_VEGFA_2_SG QuantiTech Primer Assay	QT01036861
vwf	Hs_VWF_1_SG QuantiTect Primer Assay	QT00051975
Integrin β1	Hs_ITGB1_1_SG QuantiTect Primer Assay	QT00068124
RPL13A	Hs_RPL13A_1_SG QuantiTect Primer Assay	QT00089915

## Data Availability

Data are presented in the manuscript.

## Supplementary Materials

The following supporting information can be downloaded at: https://www.mdpi.com/article/10.3390/ijms23063379/s1.
