# Supplementary material for: Graphene Oxide Framework Structures and Coatings: Impact on Cell Adhesion and Pre-Vascularization Processes for Bone Grafts
Source: Int J Mol Sci. 2022 Mar 21;23(6):3379. doi: 10.3390/ijms23063379 (PMC8955516; doi:10.3390/ijms23063379)
Supplement: Supplementary file 1 [file ijms-23-03379-s001.zip › ijms-1619624-supplementary.pdf]

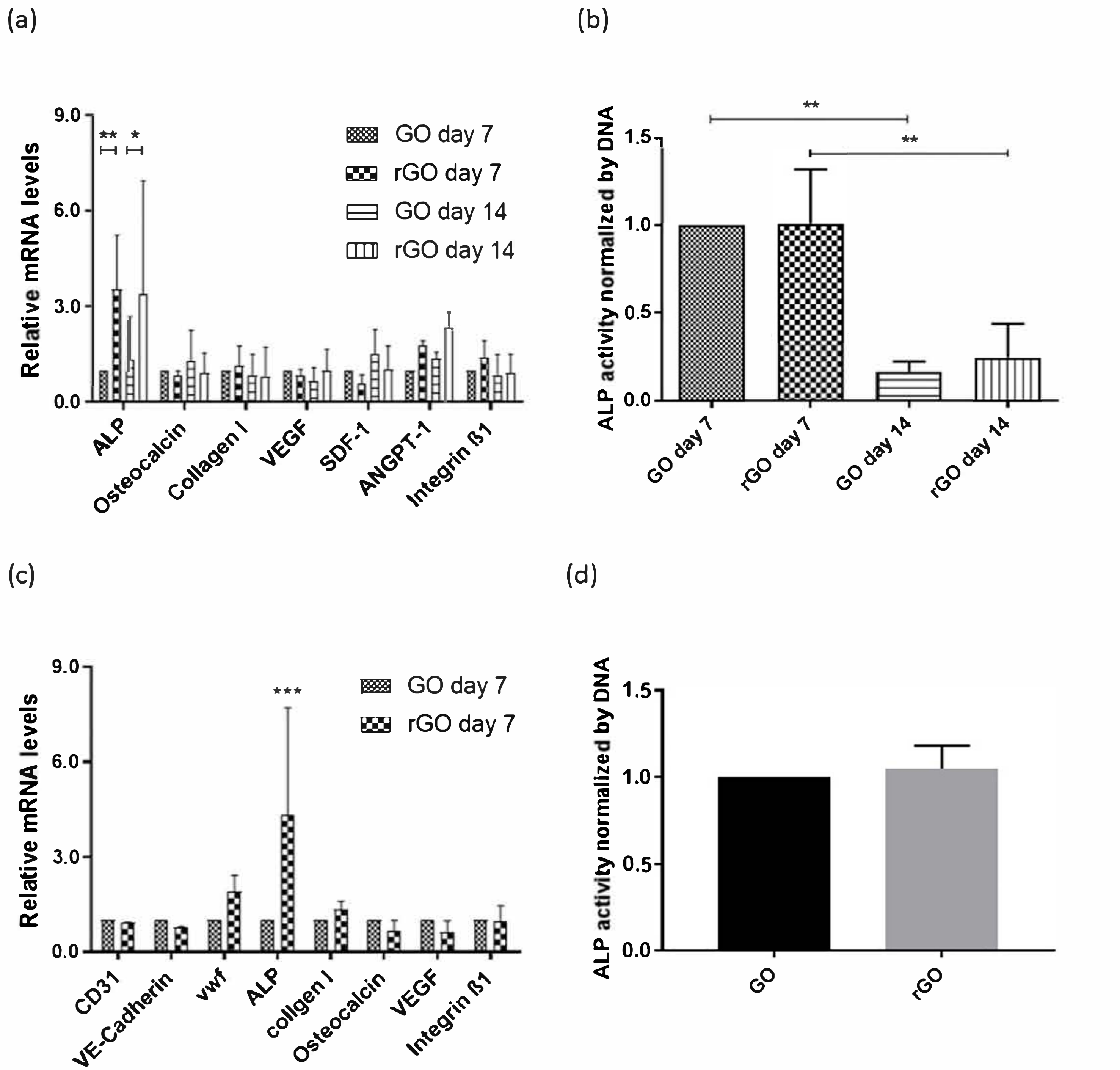

Supplementary Figure S1. (a) Relative gene expression of the osteogenic markers: ALP, osteocalcin, collagen I, angiogenesis relevant molecules: SDF-1, VEGF, ANGPT-1 and cell-matrix adhesion molecule integrin evaluated by semi-quantitative RT-PCR for MSC mono-cultures on F-GO/F-rGO on day 7 and 14. 2-way ANOVA, *$p<0.05$, n=3. (b) The ALP activity normalized by DNA content for the MSC mono-cultures on F-GO (as control) and F-rGO on day 7 and 14. 1-way ANOVA, *$p<0.05$, **$p<0.01$, n=3. (c) Relative gene expression of the endothelial, osteogenic markers and cell-matrix adhesion molecule integrin evaluated by semi-quantitative RT-PCR for MSC/OEC co-cultures on F-GO/F-rGO on day 7. 2-way ANOVA, ***$p<0.001$, n=3. (d) The ALP activity normalized by DNA content for MSC/OEC co-cultures on F-GO (as control) and F-rGO on day 7. t-test, n=3
